# Assessment of Lead Exposure and Urinary-δ-aminolevulinic Acid Levels in Male Lead Acid Battery Workers in Tamil Nadu, India

**DOI:** 10.5696/2156-9614-8.17.6

**Published:** 2018-03-12

**Authors:** Ravibabu Kalahasthi, Tapu Barman

**Affiliations:** Regional Occupational Health Centre, (Southern) Bengaluru, Karnataka, India

**Keywords:** blood lead levels, lead battery plant, BLL, urinary-δ-aminolevulinic acid

## Abstract

**Background.:**

Exposure to lead (Pb) affects multiple health outcomes and physiological systems. In adults, even small increases in blood Pb levels have been associated with decreased glomerular filtration rate, increased risk of hypertension and increased incidence of essential tremor. To date, there have been few Pb-exposure assessments using the United States Occupational Health and Safety Administration (OSHA) regulations.

**Objective.:**

The aim of the present study was to assess Pb-exposure in terms of elevated blood lead levels (BLL) and urinary-δ-aminolevulinic acid (U-δ-ALA) levels of workers exposed to Pb in the lead acid battery industry in Tamil Nadu, India based on Pb exposure regulations set by the American Conference of Governmental Industrial Hygienists (ACGIH) and OSHA.

**Materials and Methods.:**

BLLs and U-δ-ALA were estimated in 449 male workers exposed to Pb across ten different job categories in a lead acid battery factory. Worker BLLs were estimated using atomic absorption spectrophotometry and U-δ-ALA was estimated using spectrophotometry.

**Results.:**

The Biological Exposure Index of the American Conference of Governmental Industrial Hygienists (BEI-ACGIH) were used to assess Pb exposure. BLLs <30 μg/dL were found in 63.5% of workers, and 36.5% of workers had BLLs>30 μg/dL. The present study also assessed Pb exposure using OSHA regulations and found that 83.3% of workers had BLLs <40 μg/dL and 16.7% of workers had BLLs>40 μg/dL. Among these workers, 0.7% of workers had BLLs >60 μg/dL. An excessive excretion of U-δ-ALA (20–40 mg/L) was noted in pasting area workers (2.6%) followed by executives (2.2%) and assembly workers (0.9%).

**Conclusions.:**

Workers in the job categories of pasting and assembly, as well as executives, are at high risk of Pb exposure compared to other job categories. We recommend placing humidifiers on the roof and keeping a water bath closer the to plate cutting area to reduce fugitive Pb dust emissions. We recommended workers with BLLs >60 μg/dL be removed from jobs involving Pb exposure and return to work only when their BLLs are <40 μg/dL.

**Participant consent.:**

Obtained

**Ethics Approval.:**

The study was approved by the ethics committee of the Regional Occupational Health Centre (Southern) Bengaluru, part of the National Institute of Occupational Health of India.

**Competing Interests.:**

The authors declare no competing financial interests.

## Introduction

The manufacture of lead acid batteries involves several processes, such as the preparation of lead-oxide, grid casting, pasting, plate cutting, formation, charging and assembly. The chemicals used in these processes are hazardous by nature; they include lead oxide (PbO_2_), spongy lead (Pb) and sulfuric acid (H_2_SO_4_). Lead can enter the bodies of workers through inhalation and ingestion. Inhalation is the primary route of exposure in Pb-related occupations.^1^ After entry into the body, Pb accumulates in erythrocytes, soft tissue (brain, kidney, and bone marrow) and mineralized tissue (bone and teeth). Exposure to Pb can lead to hypertension, nephropathy, anemia and decreased growth. Lead affects several body systems, including the gastrointestinal, immune, and nervous system, along with behavioral/cognitive/intelligence quotient (IQ) defects in children.^2^ The monograph of the National Toxicology Program of the US Department of Health and Human Services states that even blood lead levels (BLLs) as low as <5 μg/dL are associated with adverse health effects in adults.^3^,^4^ Worker BLLs have been associated with respiratory, gastrointestinal and musculoskeletal morbidities.^5^

Reticulocytosis has also been reported in recent studies among lead acid battery manufacturing workers, along with altered genetic polymorphism of aminolevulinic acid dehydratase (ALAD) activity, a high prevalence of illnesses associated with Pb toxicity, altered neurobehavioral function, Pb-encephalopathy, disturbed calcium and phosphorus metabolism, genotoxicity, osteoporosis risk, dental erosion, functional alteration of the brain, oxidative stress and renal dysfunction.^6–19^ Chao *et al*. reported Pb-exposure in four working zones (plate manufacture, assembly, maintenance and office) in a lead acid battery plant in Taiwan.^20^ Andrade *et al.* suggested that the estimation of urinary-δ-aminolevulinic acid (U-δ-ALA) is a potential biomarker of exposure to neurotoxic metals, including Pb.^21^,^22^ Ahmed *et al.* reported significantly higher levels of U-δ-ALA in lead battery repair unit workers compared to controls.^23^

Pb-exposure inhibits three enzyme activities in heme synthesis.^24^ The affected enzymes are aminolevulinic acid synthetase, delta-aminolevulinate dehydratase (ALAD) and ferrochelatase. The estimation of U-δ-ALA is considered to be a surrogate biomarker of blood lead level in lead acid battery workers. U-δ-ALA is normally excreted in small amounts in urine, and levels increase with increased Pb exposure. The rise in the concentration of U-δ-ALA during Pb exposure is a primary function of the decreased activity of enzymes involved in the heme synthesis pathway.^25^ The United States Occupational Health and Safety Administration (OSHA) medical surveillance guidelines provide information to physicians regarding the examination and evaluation of workers exposed to Pb in four sections.^26^ Section one provides details on estimating BLLs, and its role in protecting workers from Pb exposure. Section two outlines clinical manifestations of Pb poisoning and the effects of Pb intoxication on enzymatic pathways in heme synthesis. Section three outlines the recommended exposed worker medical evaluation. Section four provides detailed information on laboratory tests available for the monitoring of exposed workers.

The present study examined the association between BLLs across job categories and the effect of Pb on the U-δ-ALA levels of workers exposed to Pb in a lead acid battery plant using Biological Exposure Index of the American Conference of Governmental Industrial Hygienists (BEI-ACGIH) (>30 μg/dL) and OSHA medical surveillance guidelines (>40 μg/dL and suspension limit is >60 μg/dL for general industry).^27^

The factory in the present study is regulated under the Indian Factories Act, 1948 (Act No. 63 of 1948), as amended by the Factories (Amendment) Act, 1987 (Act 20 of 1987)^28^ for worker health, safety and enforcement of rules for environmental regulation. The company is also registered under the Indian Companies Act, 1913. The manufactured batteries are used in automobiles and standby power applications. The Pb-battery manufacturing process is considered to be a hazardous process as per the first schedule under Section 2(cb) and Pb-poisoning is considered to be a notifiable disease as per the third schedule under Section 89 and 90 of the Indian Factories Act.^28^ The employees working in the job processes outlined in this study may be exposed to higher Pb concentrations than the recommended limits of ACGIH threshold limit values (threshold limit value–time-weighted average 50 μg/m^3^)^27^ and OSHA-permissible exposure limits (PEL 50 μg/m^3^ as an 8 hour time-weighted average and action level is 30 μg/m^3^),^27^ respectively. There have been few studies assessing Pb-exposure using BEI-ACGIH and OSHA medical surveillance guidelines among workers in India.

**Abbreviations**
*BEI-ACGIH*: Biological Exposure Index of the American Conference of Governmental Industrial Hygienists
*BLLs*: Blood lead levels
*OSHA*: Occupational Health and Safety Administration
*U-δ-ALA*: Urinary-δ-aminolevulinic acid

## Materials and Methods

The present study was carried out at a lead acid battery manufacturing plant located in Tamil Nadu, India, under the study area of the Regional Occupational Health Centre (Southern) Bengaluru.

Lead exposure was assessed in terms of elevated BLLs and U-δ-ALA among 449 male workers involved in ten different job categories: assembly, casting, pasting, ball mill, charging, plate cutting, engineering, formation, acid filling and executives. The study was approved by the ethics committee of the Regional Occupational Health Centre (Southern) Bengaluru, part of the National Institute of Occupational Health of India. The subjects were informed about the study and consent was obtained prior to their participation.

The present study used a cross-sectional survey design. The total workforce at the factory was 449 subjects, and all were men. No female workers are employed in the production areas of the factory, or as executives. The demographic information was collected from a pre-structured questionnaire and can be found in Supplemental Material. The collected information was entered into Microsoft Excel, converted into Statistical Package for the Social Sciences (SPSS) software, and the data was analyzed.

### Blood Lead Levels

Two (2) ml of venous whole blood was collected in a heparinized Vacuette from the study subjects and stored at −20°C until analysis. Using ETHOS-D, a microwave digestion system for sample preparation (Milestone S.r.l., Sorisole, Italy), 2 ml of whole blood was digested with 2 ml of nitric acid (HNO_3_) and 0.2 ml of hydrogen peroxide (H_2_O_2_) while maintaining power, temperature and duration. The digested samples were made up to 5 ml using distilled water and centrifuged. The concentration of Pb was measured using an atomic absorption spectrophotometer (GBC Avanta P, Australia). A standard solution of 20 μg/dl of Pb was prepared from the stock standard solution obtained from Merck (1.19776.0500) and added to the lowest concentration of the sample. The analysis found a 100% recovery with % relative standard deviation at <0.5 for three replicates and BLLs were expressed as μg/dl.

### Urinary-δ-aminolevulinic Acid

Urine samples were collected from study subjects using a polypropylene container. To avoid contamination, a midstream portion of the urine sample was collected in a sample container wrapped with aluminum foil for protection from sunlight exposure. The collected urine samples were preserved at 4°C until analysis. In this experiment, the internal quality control method was used. In this method, a known concentration of δ-aminolevulinic acid (δ-ALA) is added to the lowest concentration of a urine sample, with a 99% recovery. U-δ-ALA levels were determined using the method of Tomokuni and Ogata.^29^ Using this approach, the urine sample was heated with buffered ethyl-acetoacetate to produce a pyrrole derivative. This derivative was extracted into ethyl acetate and Ehrlich's reagent was added to produce a reddish color. After 10 minutes, the absorbance of the color solution was recorded at 550 nm using a spectrophotometer (Elico-SL159). Urinary-δ-ALA was calculated from a standard curve prepared using an aqueous standard solution in the range of 0 to 30 mg/L of δ-ALA. The level of U-δ-ALA was expressed as mg/L.

## Results

The demographic details of Pb-exposed workers across different job categories are reported in Table 1. The highest percentage of workers were employed in the assembly area, followed by engineering, executive positions, casting and pasting. The mean age and experience among plate cutting workers were high compared to the other job categories. The mean and 25–75th percentiles of BLLs across job categories were reported. The highest mean BLLs were found in pasting area workers, followed by plate cutting, assembly, executive positions and ball mill area workers. The BLLs of workers from the assembly, casting, pasting and plate cutting areas were significantly greater than those of workers from the charging and formation areas. Workers in the studied factory do not move between jobs.

**Table 1:** Demographic details of workers across different job categories.

**Job Categories (n)**	**Percentage (%)**	**Age (years)**	**Experience**	**Mean BLLs (μg/dL) (25–75th percentile)**
*Assembly (214)*	47.7	37.2	13.7	28.7 (21–37)
*Casting (44)*	9.8	35.7	13.7	24.1 (16–27)
*Pasting (39)*	8.7	34.4	11.9	33.2 (22–44)
*Ball mill (10)*	2.2	36.5	12.1	26.1 (16–32
*Charging (19)*	4.2	37.2	15.2	17.7 (11–22)
*Plate cutting (6)*	1.3	39.2	15.6	31.3 (25–37)
*Engineering (53)*	11.8	38.2	15.5	21.6 (11–27)
*Formation (5)*	1.1	36.4	15.2	14.0 (11–16)
*Acid filling (10)*	2.2	38.5	14.9	21.6 (11–33)
*Executives (49)*	10.9	33.6	8.9	27.4 (16–37)

The distribution of BLLs among Pb-exposed workers across different job categories is reported in Table 2. The analysis of BLLs among Pb-exposed workers was performed using a cutoff value set by the BEI-ACGIH (>30 μg/dL).^27^ A total of 63.5% of workers had BLLs <30 μg/dL and 36.5% of workers had BLLs >30 μg/dL. The highest percentage of elevated BLLs was found in workers in the plate cutting area, followed by pasting, executive and assembly positions.

**Table 2:** Distribution of BLLs in Pb-exposed Workers According to BEI-ACGIH Regulations

**Job Categories(n)**	**≤30 μg/dL (n)%**	**>30 μg/dL (n)%**
*Assembly (214)*	(127) 59.3	(87) 40.7
*Casting (44)*	(34) 77.3	(10) 22.7
*Pasting (39)*	(16) 40.0	(23) 60.0
*Ball mill (10)*	(5) 50.0	(5) 50.0
*Charging (19)*	(18) 94.7	(1) 5.3
*Plate cutting (6)*	(2) 33.3	(4) 66.7
*Engineering (53)*	(43) 81.1	(10) 18.9
*Formation (5)*	(5) 100	(0) 00
*Acid filling (10)*	(7) 70.0	(3) 30.0
*Executives (49)*	(28) 57.1	(21) 42.9
*Total (449)*	285(63.5)	164(36.5)

The distribution of BLLs among Pb-exposed workers across job categories is reported in Table 3. The distribution of BLLs among Pb-exposed workers was analyzed using OSHA guidelines.^27^ The OSHA lead standards require workers to be removed from Pb exposures when their BLLs are ≥50 μg/dL (construction industry) or 60 μg/dL (general industry) and prohibit a worker from returning to work until their BLL is <40 μg/dL. The present study found that 83.3% of workers had BLLs <40 μg/dL and 16.7% workers had BLLs >40 μg/dL. Among these workers, 0.7% of workers had BLLs >60 μg/dL, and therefore they should be removed from work involving Pb exposures and return to work only when their BLL is <40 μg/dL, following OSHA guidelines.

**Table 3:** Distribution of BLLs in Lead-exposed Workers According to OSHA Regulations

**Job Categories (n)**	**≤40 μg/dL(n)%**	**41–49 μg/dL(n)%**	**50–59 μg/dL (n) (%)**	**≥60 μg/dL (n) %**
*Assembly (214)*	(172) 80.0	(31)14.5	(10) 4.6	(1) 0.4
*Casting (44)*	(38) 86.4	(6) 13.6	(0) 0	(0) 0
*Pasting (39)*	(27) 69.3	(8) 20.5	(2) 5.1	(2) 5.1
*Ball mill (10)*	(9) 90.0	(1) 10	(0) 0	(0) 0
*Charging (19)*	(18) 94.7	(1) 5.3	(0) 0	(0) 0
*Plate cutting (6)*	(5) 83.3	(1) 16.7	(0) 0	(0) 0
*Engineering (53)*	(51) 96.2	(2) 3.8	(0) 0	(0) 0
*Formation (5)*	(5) 100.0	(0) 0	(0) 0	(0) 0
*Acid filling (10)*	(10) 100.0	(0) 0	(0) 0	(0) 0
*Executives (49)*	(39) 79.6	(9) 18.4	(1) 2.0	(0) 0
*Total (449)*	374 (83.3)	59 (13.1)	13 (2.9)	3 (0.7)

The distribution of U-δ-ALA levels in Pb-exposed workers across different job categories is presented in Table 4. The distribution of U-δ-ALA among Pb-exposed workers was assessed using the guideline of Lane *et al.* for the diagnosis of Pb poisoning.^30^ Levels of U-δ-ALA were categorized into four groups: normal (<6 mg/L), acceptable (6–20 mg/L), excessive (20–40 mg/L) and dangerous (>40 mg/L). The highest percentage of excessive U-δ-ALA levels was found in pasting area workers (2.6%), followed by executive positions (2.2%) and assembly workers (0.9%). No workers were found to have dangerous levels (>40 mg/L) of U-δ-ALA.

**Table 4:** Distribution of U-δ-aminolevulinic Acid Levels in Workers Across Job Categories

**Job Categories (n)**	**Urine-δ-ALA (Normal) <6.0 mg/L (%)**	**Urine-δ-ALA (Acceptable) 6.0–20 mg/L (%)**	**Urine-δ-ALA (Excessive) 20–40 mg/L (%)**	**Urine-δ-ALA (Dangerous) >40 mg/L (%)**
*Assembly (214)*	197 (92.1)	15 (7.0)	2 (0.9)	0 (0)
*Casting (44)*	43 (97.7)	1 (2.3)	0 (0)	0 (0)
*Pasting (39)*	35 (89.7)	3 (7.7)	1 (2.6)	0 (0)
*Ball mill (10)*	10 (100)	0 (0)	0 (0)	0 (0)
*Charging (19)*	18 (94.7)	1 (5.3)	0 (0)	0 (0)
*Plate cutting (6)*	06 (100)	0 (0)	0 (0)	0 (0)
*Engineering (53)*	52 (98.1)	1 (1.9)	0 (0)	0 (0)
*Formation (5)*	05 (100)	0 (0)	0 (0)	0 (0)
*Acid filling (10)*	10 (100)	0 (0)	0 (0)	0 (0)
*Executives (49)*	42 (85.6)	6 (12.2)	1 (2.2)	0 (0)

The association between BLLs and U-δ-ALA levels of Pb-exposed workers were assessed by using cut off values of BEI-ACGIH (>30 μg/dL) and US-OSHA regulation (>40 μg/dL). BLLs were positively and significantly associated with U-δ-ALA levels of Pb-exposed workers. The correlation coefficient of workers with BLLs >30 μg/dL was 0.245 (P=0.002), and the correlation coefficient of workers with BLLs >40 μg/dL was 0.375 (P=0.001).

## Conclusions

The present study estimated BLLs and U-δ-ALA in workers exposed to Pb at a lead acid battery plant to assess Pb exposure under Sections 1 and 2 of the OSHA medical surveillance guidelines.^26^

Pb-exposure was also assessed using the guidelines of the BEIACGIH. 27 The present study found that 63.5% of workers had BLLs <30 μg/dL and 36.5% of workers had BLLs >30 μg/dL. Were FH *et al.* reported that 100% of lead battery manufacturing workers had BLLs >30 μg/dL, with a mean airborne Pb concentration of 133μg/m^3^.^31^ All of the measured concentrations of airborne Pb exceeded the OSHA permissible exposure limit of 50 μg/m^3^. Lead-acid battery manufacturing plants in developing countries were found to have an average worker BLL of 47μg/dL and an average airborne Pb concentration of 367 μg/m^3^, which is 7-fold higher than the OSHA permissible exposure limit.^32^ Ravichandran *et al.* reported mean airborne Pb concentrations in plate buffing, plate cutting, pasting, assembly, oxide mill and casting areas in a lead-acid battery manufacturing plant in India of 1444, 430, 277, 77, 63 and 11 μg/m^3^, respectively.^33^ The highest percentages of elevated BLLs (>30 μg/dL) were noted in plate buffing workers, followed by plate cutting, pasting, assembly, oxide mill and casting. In the present study, we noted the highest percentage of elevated BLLs (>30 μg/dL) in plate cutting workers followed by pasting, ball mill and assembly positions. Kalahasthi *et al.* reported the highest percentage of elevated BLLs (>30 μg/dL) in pasting workers (74%), followed by oxide mill (44%), assembly (40%) and plate cutting (25%) area workers in India.^34^ As per the noted airborne and blood Pb concentrations in lead-acid battery manufacturing plants, the job categories of pasting, plate cutting and assembly sections presented the high risk for Pb-exposure.

In the present study, we noted that 83.3% of workers had BLLs <40 μg/dL and 16.7% of workers had BLLs higher than OSHA recommended limits of >40 μg/dL. Chao *et al.* found that 63.3% of workers had BLLs <40 μg/dL and 36.7% of workers had BLLs >40 μg/dL in workers in 23 registered Taiwanese lead-acid battery manufacturing plants.^20^ The high results may be because the study was conducted in 1992. Ahmad *et al.* found that 84% of Bangladesh lead-acid battery manufacturing workers had BLLs > 40 μg/dL, 5 times higher than the present study, attributed to a poor working environment, inadequate use of personal protective equipment (PPE) and long work shifts.^9^ Lormphongs *et al.* assessed the BLLs in workers from a lead-acid battery plant in Thailand from 1998–2002.^35^ The rate of BLLs >40 μg/dL among Thai workers ranged from 25.1% in 1999 to 7.7% in 2002. The BLLs of some workers exceeded the threshold level (>60 μg/dL) of the Ministry of Public Health of Thailand (2.1% in 1998, 1.1% in 1999, 0.6% in 2000 and 2.7% in 2001). In the present study, 0.7% of workers had BLLs >60 μg/dL. In the United Kingdom, one study reported that 0.6% of workers had BLLs >60 μg/dL, which is the suspension limit.^36^ Matte *et al.* reported that 28% of Jamaica lead-acid battery workers had BLLs >60 μg/dL.^37^ The authors concluded that the factory had inadequate controls and respiratory protections.

The estimation of U-δ-ALA levels is used as a diagnostic tool to determine possible Pb poisoning.^38^ Kalahasthi *et al.* reported a positive association between BLLs and U-δ-ALA levels with high levels of lead exposure (>30 μg/dL) compared to low levels of exposure (<30 μg/dL).^34^ Makino *et al.* also assessed the relationship between BLLs and U-δ-ALA levels and reported that U-δ-ALA levels were decreased at BLLs lower than 20 μg/dL.^39^ Wang et *al.* reported no association between low levels of Pb exposure (mean BLLs 6.7 μg/dL) and U-δ-ALA.^40^ In the present study, there was a positive and significant association noted between BLLs and U-δ-ALA levels for workers with BLLs <40 μg/dL(r=0.143, P=0.006) and >40 μg/dL (r=0.375, P=0.001). The higher correlation coefficient (r) was noted in workers with BLLs >40 μg/dL compared to BLLs <40 μg/dL. These studies did not assess Pb exposure using the Lane *et al.* classification for U-δ-ALA.^30^ U-δ-ALA levels were classified into four groups: normal (<6 mg/L), acceptable (6–20 mg/L), excessive (20–40 mg/L) and dangerous (>40 mg/L). Ahmed *et al.* estimated U-δ-ALA levels in workers from lead acid battery repair units and noted that over 50% of workers had an acceptable limit and one-third of workers had excessive levels (20–40 mg/dL).^23^ In the present study, 85.6% of workers had normal U-δ-ALA levels, 12.2% of workers had acceptable U-δ-ALA levels, and 2.2% of workers had excessive U-δ-ALA excretions. In the excessive urinary-δ-ALA excretion category, the highest percentage was observed in pasting area workers, followed by those working in executive positions and assembly. The findings of high levels of U-δ-ALA among pasting area workers is an indication of excessive Pb exposure. During the pasting process, Pb-oxide paste is applied to the grid panels in the pasting machine to fill the spaces of the grid, which are then mechanically dried in a hot air oven. In this process, the Pb-oxide coating becomes airborne. For this reason, the workers in the pasting area are at greater risk of Pb exposure compared to other job categories. These workers are also exposed to Pb through contaminated gloves, clothing and equipment. The BLLs among executives were found to be elevated, perhaps because executives work in shop floor areas and spend most of their time with workers supervising operations, and therefore have similar exposures to other plant workers. During a visit to the work site, we noted that all of the workers and executives were wearing PPE such as hand gloves, respiratory masks, uniforms and safety shoes. The workers were not permitted to take the uniforms home. In addition to the use of PPE, additional control measures are needed to minimize occupational Pb exposure.

## Recommendations

The present study assessed Pb exposure using BLLs and U-δ-ALA levels in workers exposed to Pb at a lead acid battery plant. A total of 16.7% of workers had BLLs >40 μg/dL. The highest BLLs and U-δ-ALA levels were found in pasting area workers. Based on observations and lead acid battery industry work practices, we suggest placing humidifiers on the roof and keeping water baths in the plate cutting area in order to minimize fugitive Pb dust emissions. We also recommend that workers with BLLs>60 μg/dL be removed from work involving Pb exposure and return to work only when their BLLs are <40 μg/dL following OSHA guidelines.
