# Supplementary material for: Assessment of Lead Exposure and Urinary-δ-aminolevulinic Acid Levels in Male Lead Acid Battery Workers in Tamil Nadu, India
Source: J Health Pollut. 2018 Mar 12;8(17):6–13. doi: 10.5696/2156-9614-8.17.6 (PMC6221436; doi:10.5696/2156-9614-8.17.6)
Supplement: Supplementary file 1 [file i2156-9614-8-17-6.s1.pdf]

Regional Occupational Health Centre (Southern)

ICMR Complex, Kannamangala PO, Poojanahalli Road

Devanahalli Taluk, Bengaluru-562110, Karnataka, INDIA.

Biological Monitoring of Lead Exposure in the Work Place

DEMOGRAPHIC DETAILS

1. Name: ____
2. Sex: Male / Female
3. Date of Birth: _______________________________
4. Height: __________________(cm)
5. Weight _________________(kg)
6. Department: __________________________________
7. Job title with short description:____
   ____
8. Number of years employed in this occupation: _________________
9. Previous occupation if any: ____
10. Number of years employed in previous occupation: ______________
11. Residential address with contact phone number:_____________________________________
    ____
12. Highest grade of education completed; ____
13. Do you currently smoke tobacco or have you smoked tobacco in the last month: Yes / No

    If yes

    a) No of cigar or beedies you smoke per day _______

14. Do you drink alcohol: Yes / No
    a) If yes Daily / Weekly / occasional
    b)
